# An Account of the Efficacy of Sugar of Lead in Curing Epilepsy

**Published:** 1805-07-01

**Authors:** Benjamin Rush

**Affiliations:** Philadelphia


*An Account of the Efficacy of Sugar of Lead in curing Epilepsy. By* BENJAMIN RUSH, M. D. *of Philadelphia.*

ABOUT four years ago, Mrs. Toy, of Southwark, brought her son to me, a boy of ten years old, who, she said, had been afflicted for some time with epileptic fits. I prescribed for him a pill of two grains of the acetate, commonly called sugar of lead, three times a day. In a few months it perfectly cured him, and I had the pleasure of hearing a few months ago, that he has continued ever since to enjoy good health.

In the year 1802, I cured a boy of Mr. Robert E. Hobart of the same disease, by the use of the same dose of the same remedy. This boy was of a full habit. Previously to his taking the medicine, I drew eight ounces of blood from his arm, in order to increase the effect of the medicine, agreeably to the practice of Dr. Sydenham in a similar disease.

I am now administering it to a boy of Mr. Adam Stricker, aged twelve years, in a low grade of epilepsy which had affected him for some months. It has prevented a return of his fits nearly two months; and I am disposed to believe it will finally cure him.

I have used the sugar of lead in several cases of epilepsy in adults. In one of them it suspended the fits for several weeks, but I am sorry to add, in no case did it perform a cure.

The epilepsy is not singular in yielding to medicines under puberty, which have little or no effect in adult age. Pulmonary consumption, chronic eruptions upon the head, scrophula, the chorea sancti Viti, and several other diseases, are often cured under puberty by remedies, which are ineffectual after the one-and-twentieth year of life.

Considering how often epilepsy, and other convulsive diseases, make their first appearance in childhood, and under puberty, it is consoling to reflect that we are possessed of a medicine which promisss to be useful in them.

Lest it should be supposed, the change which puberty makes in the system concurred in the cure of the above cases, it may not be amiss to add, that two of the subjects of them were under twelve, and the other between twelve and thirteen years of age.

I was first led to prescribe the sugar of lead in the epilepsy, by hearing that a man had been cured of it by swallowing part of a table spoonful of white lead by mistake, instead of a table spoonful of loaf sugar.

August 2, 1804.

